# From mobile phone screen to taste buds: A study on the impact of Douyin live streaming E-service scenarios on Durian consumer trust and purchase intention

**DOI:** 10.1371/journal.pone.0351421

**Published:** 2026-08-19

**Authors:** Xuemei Xu, Anita Rosli, Aryaty Alwie, Ismawati Sharkawi, Yurong Wu

**Affiliations:** 1 Faculty of Business, ChongQing College of Humanities, Science and Technology, ChongQing, China; 2 Department of Social Science and Management, Faculty of Humanities, Management and Science, Universiti Putra Malaysia Sarawak, Bintulu, Sarawak, Malaysia; 3 Department of Recreation and Ecotourism, Faculty of Forestry and Environment, Universiti Putra Malaysia, Serdang, Selangor, Malaysia; MCC Boyd Tandon School of Business, INDIA

## Abstract

While live streaming has transformed e-retailing, its application to high-value, perishable agricultural products remains under-theorized. This study aims to explore the micro-mechanism of digital marketing for high-value regional products,reshape the e-servicescape framework specifically for fresh durians sold on social commerce platforms (Douyin), examining the psychological mechanisms driving impulse buying. Adopting an exploratory sequential mixed-methods design, the research first conducted in-depth interviews with 18 consumers, employing the Gioia methodology to inductively identify unique agricultural environmental cues. Subsequently, a quantitative survey of 428 verified buyers was analyzed using Partial Least Squares Structural Equation Modeling (PLS-SEM) to empirically test the proposed Stimulus-Organism-Response (S-O-R) model. The qualitative findings enriched the e-servicescape by identifying four distinct dimensions: Visual Freshness (diagnostic quality cues), Streamer Expertise (quality gatekeeping), Real-time Interactivity (social presence), and Rural Support Consciousness (altruistic motivation). The quantitative results confirmed that Visual Freshness and Streamer Expertise significantly influence impulse buying through the full mediation of Perceived Trust, whereas Real-time Interactivity and Rural Support Consciousness drive behavior via Perceived Enjoyment. Uniquely, the study validates that moral satisfaction from supporting farmers acts as a hedonic driver in this context. These findings not only advance theoretical understanding by contextualizing fresh fruit marketing but also offer a validated digital-intelligence driven path for upgrading regional industries, providing strategic references for the digital transformation of cultural and agricultural tourism sectors.

## 1. Introduction

The advent of live streaming commerce has fundamentally revolutionized the global retail landscape, transforming static online shopping into a dynamic, interactive, and immersive experience [1]. This transformation is particularly evident in China, where platforms like Douyin (Chinese TikTok) have pioneered interest e-commerce, integrating entertainment with instant purchasing [2]. Against the backdrop of the Digital China strategy, regional industries are actively seeking digital-intelligence driven development paths to revitalize local economies. Recent literature emphasizes that digital transformation in regional sectors extends beyond mere technological adoption; it fundamentally restructures value creation and consumer engagement [3]. Within this digital shift, live streaming commerce disrupts consumers’ habitual decision-making processes, creating new pathways for immediate behavioral responses [4].This aligns directly with ongoing initiatives exploring the practice of Chinese modernization within the digital economy of regions like Guizhou, which possesses rich cultural and agricultural resources but faces challenges in connecting with broader markets.While the impact of live streaming on fashion and cosmetics has been extensively studied [5,6], its application in the agricultural sector,specifically for high value, perishable fresh produce,presents a unique and under-researched phenomenon [7].Understanding this mechanism is crucial not only for the specific product category but also for providing empirical evidence for broader initiatives, such as the Pathways of Digital-Intelligence Driven Development, which aims to leverage digital tools to promote high-value regional offerings.

Among agricultural products, the durian often hailed as the King of Fruits,has emerged as a viral sensation in live streaming social commerce [8]. Unlike standardized manufactured goods, fresh durians are characterized by high unit prices, significant heterogeneity (e.g., varying ripeness and flesh yield), An attribute where the internal quality remains unknown until the shell is opened [9].These characteristics high value, high experience dependence, and non-standardization,create significant information asymmetry and perceived risk for online consumers. These characteristics create high information asymmetry and perceived risk for online consumers [10]. However, live streaming seems to mitigate these barriers effectively through real-time visual demonstration and streamer interaction, triggering frequent impulse buying behaviors [11]. Despite this practical boom, the theoretical mechanism underlying how the live streaming environment drives impulse purchases for such high-risk agricultural products remains unclear.

The concept of e-servicescape,the online environmental cues that influence customer responses,has been widely adopted to explain online consumer behavior [12]. Recent studies have attempted to extend this concept to the live streaming context [13,14]. Recently enriched the retail e-servicescape by identifying dimensions such as attractiveness and authenticity through a qualitative lens [15]. However, existing frameworks largely focus on general retail settings and fail to capture the nuances of agricultural marketing, such as the crucial role of visual freshness or the altruistic motivation of rural support [16,17]. Furthermore, There is a pressing need for quantitative empirical validation of these enriched e-servicescape dimensions to generalize findings [15].

## 2. Literature review

### 2.1. Theoretical framework: The S-O-R paradigm

To investigate the psychological mechanism driving impulse buying of fresh durians in the dynamic environment of live streaming, this study adopts the Stimulus-Organism-Response (S-O-R) framework originally proposed [17]. Rooted in environmental psychology, this paradigm posits that environmental cues (Stimulus) act as external triggers that induce internal emotional and cognitive states (Organism), which subsequently lead to approach or avoidance behavioral responses (Response).

#### 2.1.1. Evolution of S-O-R in E-commerce.

Historically, the S-O-R model was applied to physical retail settings to understand how store atmospherics (e.g., lighting, music) influenced shopper behavior [18]. With the advent of the internet, Eroglu et al. (2001) adapted this framework to the online context, verifying that screen-based cues could similarly evoke psychological reactions [19]. In recent years, the model has been extensively validated as a robust lens for understanding consumer behavior in social commerce and live streaming contexts [20,21].

#### 2.1.2. Application in live streaming.

The strength of the S-O-R framework lies in its ability to unbox the black box of consumer decision-making. In live streaming commerce, the Stimulus is no longer a static webpage interface but comprises the live streaming e-servicescape,a complex, synchronized mix of visual product displays, auditory streamer pitches, and real-time social interactions [22]. The Organism represents the consumer’s internal psychological processing, which acts as a critical mediator. This is typically categorized into cognitive reactions, such as the evaluation of credibility and trust, and affective reactions, such as the feeling of enjoyment or flow [13]. Finally, the Response in this study manifests as the urge to purchase impulsively, a behavior characterized by suddenness and a lack of strict planning [23]. This study applies this framework to the unique context of agricultural e-commerce, positing that platform-specific environmental cues reshape consumer psychology regarding high-risk fresh produce.

### 2.2. Live streaming E-servicescape in agriculture

#### 2.2.1. The concept of E-servicescape.

The concept of servicescape, originally coined by Booms and Bitner (1981), refers to the man-made physical environment where service encounters take place [24]. Harris and Goode (2010) extended this to the e-servicescape, defining it as the online environmental factors that exist during service delivery [12]. While early research focused on static website attributes like aesthetic appeal, layout efficiency, and financial security [25], recent scholarship has shifted towards the dynamic, synchronized environment of live streaming [1]. In live streaming, the servicescape is transient, immersive, and socially constructed, heavily relying on the streamer’s performance and the immediate visual context.

#### 2.2.2. Contextualizing for agricultural products.

Teng and Balakrishnan (2025) recently enriched this domain by identifying dimensions such as attractiveness, authenticity, and community consciousness in general retail live streaming [15]. However, agricultural products,specifically non-standardized fruits like durians,require distinct environmental cues that differ significantly from standardized manufactured goods.

Durians are characterized by high unit prices and significant quality heterogeneity (e.g., variances in flesh yield, taste, and ripeness), creating a classic lemon market problem plagued by high information asymmetry [7,26]. Unlike apparel or electronics, fresh produce relies heavily on sensory attributes. In the absence of touch and smell online, visual freshness becomes a critical proxy for quality [10]. Furthermore, the rising trend of digital agriculture and rural revitalization in China has introduced altruistic motivations into the shopping process. Thus, this study argues for a reshaped agricultural e-servicescape that prioritizes sensory-enabling cues (to prove quality) and altruistic signaling (to prove social value) over mere aesthetic appeal.

### 2.3. Hypothesis development

#### 2.3.1. Agricultural E-servicescape stimuli and perceived trust.

Trust is defined as the belief that an online seller will behave benevolently and competently, which is a critical precursor to transaction intention in uncertain online environments [1]. For durians, a product characterized by blind box uncertainty,where the internal quality is unknown until the shell is opened,trust is the primary barrier to purchase [8].

Visual Freshness.In traditional e-commerce, consumers rely on static images which can be easily manipulated. However, in live streaming, high-definition, real-time visual presentation serves as the primary diagnostic cue for product quality [16]. According to Product Diagnosticity Theory, when consumers cannot physically inspect a product, they rely on intrinsic cues that are most indicative of quality. For durians, visual cues such as the golden color of the flesh, the texture when pressed by the streamer, and the thickness of the shell are critical. A vivid, unfiltered display of these attributes reduces perceived risk and information asymmetry, thereby enhancing cognitive trust.

Streamer Expertise.Source Credibility Theory suggests that an endorser’s effectiveness depends on their expertise and trustworthiness [27]. In the niche market of fruit retailing, the streamer acts as an opinion leader and a quality gatekeeper. A streamer’s ability to professionally identify ripeness (e.g., the technique of knocking on the shell to listen for a hollow sound) and distinguish between cultivars (e.g., Musang King vs. Monthong) signals professional competence [28]. This demonstrated expertise reassures consumers that the product has been curated by a knowledgeable agent, effectively transferring trust from the person to the product.

#### 2.3.2. Agricultural E-servicescape stimuli and perceived enjoyment.

Perceived enjoyment reflects the hedonic value of the shopping experience, defined as the extent to which the activity of using the computer is perceived to be enjoyable in its own right [5]. Live streaming transforms utilitarian grocery shopping into shoppertainment.

Real-time Interactivity.The defining feature of live streaming is its synchronicity. Real-time interactivity allows for immediate feedback loops between the streamer and the audience. This responsiveness creates a sense of Social Presence, making the virtual interaction feel warm and human [14]. When streamers respond to specific requests (e.g., open that round one for me), it enhances the consumer’s sense of control and immersion. This fluid interaction fosters a state of Flow, significantly increasing the perceived enjoyment and playfulness of the shopping episode [29].Moreover, recent studies on feelings-oriented digital behavior suggest that affect and emotional states during smartphone use are deeply intertwined with user engagement [30]. The synchronized communication in live streaming enhances empathetic connections between the streamer and the audience, which is a critical mechanism for bridging psychological distance and fostering situational trust and enjoyment [31].

Rural Support Consciousness.Extending the community consciousness dimension identified by Teng and Balakrishnan (2025), this study proposes a dimension specific to agricultural contexts: Rural Support Consciousness. This refers to the consumer’s awareness that their purchasing behavior directly supports farmers or rural revitalization. According to the Warm-glow theory of pro-social behavior, individuals derive utility and emotional satisfaction from the act of giving or helping others [32]. In agricultural live streaming, witnessing the direct link between the purchase and the farmer’s livelihood (e.g., broadcasting from the orchard) generates moral satisfaction, which contributes to the overall psychological enjoyment of the shopping process.This aligns with recent findings on digital morality, which assert that consumers increasingly evaluate social-functional attitudes and moral fulfillment during transactions [33]. Consequently, the integration of agricultural origin authenticity provides consumers with profound emotional fulfillment that transitions their online interaction into a meaningful offline socio-economic contribution [34].

#### 2.3.3. The organism-response relationship: Trust, enjoyment, and impulse buying.

Impulse buying is defined as a sudden, compelling, hedonically complex purchasing behavior in which the rapidity of the impulse decision precludes thoughtful consideration [35,36].

While impulse buying is often associated with irrationality, for high-ticket perishable items like durians, a baseline of cognitive trust is a prerequisite. High perceived trust reduces the cognitive burden of risk assessment [6]. When consumers trust the quality guarantee (e.g., bad fruit refunds), the psychological barrier to immediate action is removed. Trust functions as a safety net that allows the consumer to yield to the impulse trigger without the paralyzing fear of financial loss.

Positive affect is a powerful driver of impulse behavior. When consumers experience enjoyment and flow, their psychological guarding mechanisms are relaxed. The Broaden-and-Build theory suggests that positive emotions broaden an individual’s thought-action repertoire [10]. In a state of high enjoyment, consumers are more likely to engage in exploratory behaviors and seek immediate gratification. The entertainment value of the live stream distracts from the pain of payment, leading to higher impulse buying intention (**S1 Fig**) [23].

## 3. Methodology

### 3.1. Research design

To comprehensively investigate the reshaping of the agricultural e-servicescape and its impact on impulse buying, this study adopts an exploratory sequential mixed-methods design [37]. This approach is characterized by a two-phase structure: qualitative data collection and analysis followed by quantitative data collection and analysis.

The rationale for this design is twofold. First, the existing e-servicescape frameworks are largely derived from general retail contexts and may not fully capture the nuances of live streaming agricultural marketing, such as the unique sensory cues required for durians (e.g., visual freshness) or the altruistic motivations involved (rural support) [12,15]. Therefore, a qualitative phase is necessary to explore and identify these context-specific dimensions (Stimuli). Second, to ensure the generalizability of these findings, a subsequent quantitative phase is required to empirically validate the relationships within the proposed S-O-R model (Ying et al., 2021). The integration of both methods provides a more robust understanding than either method alone.

### 3.2. Phase 1: Qualitative study

The primary objective of the first phase was to identify the specific environmental cues (Stimuli) that trigger consumer trust and enjoyment in the context of live streaming for fresh durians.

#### 3.2.1. Data collection.

Sampling Strategy:Purposive sampling was employed to recruit participants who possessed relevant experience and insights [38]. The inclusion criteria were: (1) having purchased fresh durians via Douyin live streaming at least twice in the past three months; (2) being aged between 18 and 45, representing the primary demographic of social commerce users; and (3) willingness to articulate their shopping experiences.

Procedure:Semi-structured in-depth interviews were conducted with 18 participants during July 2025. The sample size was determined by the principle of theoretical saturation, where data collection ceased when no new themes emerged [37]. The interview protocol was grounded in the S-O-R framework, using critical incident techniques to probe specific moments of impulse buying (e.g., Recall a moment when a streamer’s action made you buy immediately. What exactly did you see or hear?). Interviews lasted approximately 30–45 minutes and were recorded and transcribed verbatim.

#### 3.2.2 Data analysis (Gioia Methodology).

To ensure rigor in inductive theory building, the data were analyzed using the Gioia Methodology [39]. This systematic approach involved three steps of coding:

1st Order Analysis: Open coding was performed on the transcripts to identify initial concepts using the participants’ own language (in vivo codes), such as golden flesh color or knocking on the shell.

2nd Order Analysis: Similar concepts were aggregated into theoretical themes. For instance, close-up of pulp and shell thickness demonstration were collapsed into the theme Visual Freshness Cues.

Aggregate Dimensions: The themes were further distilled into the core

constructs of the research model, specifically defining the dimensions of the Agricultural Live Streaming E-Servicescape (Visual Freshness, Streamer Expertise, Real-time Interactivity, and Rural Support Consciousness).

To ensure reliability, two researchers coded the data independently, achieving an inter-coder reliability of 92%. Discrepancies were resolved through discussion.

### 3.3. Phase 2: Quantitative study

The second phase aimed to empirically test the conceptual model and hypotheses (H1-H6) developed based on the qualitative findings and literature review.

#### 3.3.1. Instrument development.

A survey instrument was developed comprising three sections.

Section 1 (Stimulus): Measurement items for the agricultural e-servicescape were generated from the qualitative findings in Phase 1 and adapted from relevant literature. For example, Visual Freshness items were adapted from Park et al. (2021) but modified to specifically mention durian attributes (e.g., The live stream clearly showed the texture of the durian flesh) [40].

Section 2 (Organism & Response): Scales for Perceived Trust were adapted from [41], Perceived Enjoyment from Koufaris (2002), and Impulse Buying Intention from Rook and Fisher (1995) [35,42]. All items were measured on a 7-point Likert scale ranging from 1 (Strongly Disagree) to 7 (Strongly Agree).

Section 3: Demographic information including age, gender, income, and purchase frequency.

To transparently illustrate the exploratory sequential design, The details how the inductive qualitative themes (from Phase 1) were operationalized into the quantifiable constructs and specific survey items (for Phase 2). The measurement items for the agricultural e-servicescape were uniquely developed by blending the in vivo expressions of interviewees with established scales from existing literature, ensuring both ecological validity and theoretical grounding (**S1 Table**).

A pilot test was conducted with 40 respondents to assess the clarity and wording of the items, resulting in minor modifications to the Rural Support scale to better fit the Chinese context.

#### 3.3.2. Sampling and data collection.

Target Population: The target population for this study was strictly defined as consumers who had completed a purchase of fresh durians via live streaming on Douyin (TikTok China). This specific focus ensures that the respondents have engaged in the actual decision-making process within the agricultural e-servicescape, thereby increasing the ecological validity of the study.

Data Collection Procedure: To access this specific group of verified buyers, a convenience sampling method was combined with a screening mechanism. The survey was distributed through two primary channels on the Douyin platform:

Live Streaming Fan Groups: The questionnaire link was disseminated in the official fan groups (WeChat/Douyin groups) of three top-tier fruit live-streamers specializing in durian sales. These groups consist of highly active consumers who are regular viewers.Post-Purchase Interaction: Invitations were sent via direct message (DM) to users who had recently purchased durians from partnered merchants, soliciting their feedback on their shopping experience.Screening and Verification Mechanism: To rigorously verify the purchase history and ensure data quality, a two-step screening mechanism was implemented:

Mandatory Screening Question: A filter question was placed at the beginning of the survey: Have you purchased fresh durians through a Douyin live stream in the past 3 months? Only respondents who answered Yes were permitted to proceed.Purchase Frequency Validation: Respondents were asked to indicate their frequency of watching fruit live streams and purchasing durians. Inconsistent responses (e.g., Never watch but Buy daily) were flagged for removal during data cleaning.

Sample Size: Based on the 10-times rule for PLS-SEM (effect size f^2^ = 0.15), from August to December in 2025,atotal of 500 questionnaires were distributed [43,44]). After removing incomplete responses and those who failed attention checks (e.g., answering Strongly Agree to reverse-coded questions), 428 valid responses were retained for analysis, yielding an effective response rate of 85.6%.

#### 3.3.3. Data analysis strategy.

To address potential Common Method Variance (CMV) arising from survey data, this study employed two procedural remedies post-hoc. First, Harman’s single-factor test was utilized because it is a robust initial diagnostic tool to ensure no single dominant factor accounts for the majority of the variance. Second, inner model Variance Inflation Factor (VIF) values were assessed, as VIFs below the threshold of 3.3 efficiently confirm that the model is free from problematic pathological collinearity, which is a strong indicator of minimal CMV impact in PLS-SEM context [45].

Partial Least Squares Structural Equation Modeling (PLS-SEM) was selected for data analysis using SmartPLS 4 software. The choice of PLS-SEM over covariance-based SEM (CB-SEM) is justified for three reasons:Exploratory Nature: The study involves new theoretical extensions (reshaped e-servicescape dimensions) where the theory is not yet fully developed.Non-Normal Data: Impulse buying data often exhibits non-normal distribution, which PLS-SEM handles robustly compared to CB-SEM [44].Complexity: PLS-SEM is superior in handling complex models with many structural path relationships and mediators [46].

The analysis followed a two-step procedure:

Measurement Model Assessment: Evaluating internal consistency (Cronbach’s alpha, Composite Reliability), convergent validity (Factor Loadings, AVE), and discriminant validity (Fornell-Larcker Criterion, HTMT ratio).Structural Model Assessment: Examining the path coefficients (β), coefficient of determination (R2), predictive relevance (Q2), and mediating effects via bootstrapping (5,000 subsamples). Common Method Variance (CMV) was also assessed using Harman’s single-factor test and VIF values [32].

## 4. Results

This chapter presents the findings in two sequential phases. First, the qualitative results reveal the specific dimensions of the agricultural live streaming e-servicescape and delineate the psychological processes involved. Second, the quantitative results empirically validate the proposed research model using PLS-SEM.

### 4.1. Phase 1: Qualitative data and analysis

#### 4.1.1. Data collection and coding.

In-depth interviews were conducted with 18 consumers who had purchased fresh durians via Douyin live streaming. Participants were recruited through purposive sampling to ensure they possessed information-rich experiences regarding impulse buying in agricultural live streaming [38]. The interviews were conducted in Mandarin, audio-recorded, and transcribed verbatim, resulting in a total of 128 pages of text data.

The conducted a rigorous three-stage coding process using NVivo 12 software. In the first stage (Open Coding), we carefully reviewed each transcript to familiarize ourselves with the content and extracted initial codes representing fundamental elements of the raw text (e.g., looking at the shell thorns, streamer knocking on the fruit). In the second stage (Axial Coding), these initial codes were re-assessed, sorted, and combined to derive a manageable number of categories (e.g., Diagnostic Textures, Skillful Screening). In the third stage (Selective Coding), categories were further aggregated to form theoretical themes (Aggregate Dimensions) that constitute the agricultural e-servicescape [39].

To further ensure theoretical saturation, we followed the procedure suggested We split the data analysis into two phases: First, 12 transcripts were coded following the three-stage process, revealing the primary categories. Then, we coded the remaining 6 transcripts to determine if they offered new insights. During this process, we noted recurring instances of identified themes but no new substantial categories emerged. Therefore, we concluded that theoretical saturation had been reached [47,48].

During the coding process, we adopted investigator triangulation to control for subjective bias. Two authors independently coded the transcripts to generate two sets of codes. Recursive checks and refinements proceeded until all emerging themes exhibited high internal homogeneity. This agreed on approximately 92% of the codes, indicating a high level of inter-coder reliability. Discrepancies were resolved through negotiation until a consensus was reached.

Since the interviews were conducted in Chinese, we employed the translation/back-translation technique to ensure accuracy [49]. The coding scheme was translated into English by one author and then back-translated by a bilingual scholar to verify semantic equivalence.

#### 4.1.2. Qualitative findings.

The coding process generated a total of 248 reference points related to the research topic. Tthe analysis identified four distinct dimensions of the Agricultural E-Servicescape (Stimulus) and confirmed the presence of psychological mechanisms (**S2 Table**).

The results highlight that Visual Freshness (31.45%) and Streamer Expertise (25.81%) are the most frequently mentioned determinants, reflecting the high perceived risk associated with buying durians. Rural Support Consciousness (14.11%) emerged as a significant, context-specific dimension, validating its inclusion in the subsequent quantitative model (**S3 Table**).

#### 4.1.3. Detailed qualitative findings and hypothesis development.

Based on the coding scheme, this section elaborates on the identified dimensions and illustrates how these environmental cues shape consumer psychology, leading to the formulation of specific hypotheses.

1 Visual Freshness as a Diagnostic Cue for Trust

Most participants mentioned that their decisions to purchase durians were primarily influenced by visual uncertainty. Given that durians are blind box products where internal quality is hidden, consumers heavily rely on high-definition visual cues as proxies for freshness [16]. This pattern reflects a reality wherein visual details serve as the first line of defense against the risk of receiving dead (unripe) or watery fruit.For example, participants emphasized the importance of seeing specific textures, such as the softness of the thorns or the matte finish of the shell, before trusting the product. A male participant (Participant 04) recalled how these micro-visuals influenced his judgment:

I don’t care if the studio is pretty. I watch for the moment he presses the shell. If the thorn is soft and distinct, and the skin has that specific matte finish, I know it’s a ‘Grade A’ fruit. That visual detail is my signal to buy.

This finding suggests that in agricultural live streaming, visual freshness replaces the tactile experience of traditional markets. When consumers can clearly diagnose the quality through the screen, their anxiety diminishes, and cognitive trust is established. This substantiates the following Hypothesis:

Hypothesis 1: Visual freshness in live streaming has a positive impact on consumers’ perceived trust in fresh durian products.

2 Streamer Expertise as a Gatekeeper of Quality

Even after visual cues are assessed, consumers sometimes encounter a subsequent level of uncertainty regarding the internal yield (flesh content). Participants frequently mentioned that they relied on the streamer’s professional skills to screen the fruit for them. This aligns with Source Credibility Theory [27], where the endorser’s expertise transfers trust to the product.For example, when streamers demonstrated the technique of knocking on the shell to listen for a hollow sound (indicating ripeness), it acted as a powerful signal of competence. Participant 12 explained her reliance on this expertise:

The streamer knew how to listen to the sound. He knocked on ten durians and rejected eight, saying they were ‘raw water’ fruit. That professional screening made me believe his recommendation immediately.

Furthermore, the willingness to publicly reject inferior products (candid rejection) was seen as a strong indicator of integrity. This behavior mitigates the information asymmetry inherent in the lemon market of fresh produce, leading to the following Hypothesis:

Hypothesis 2: Streamer expertise has a positive impact on consumers’ perceived trust.

3 Real-time Interactivity and the ‘Flow’ of Enjoyment

While trust addresses the cognitive barriers, participants also highlighted the hedonic value of the live streaming experience. The synchronous nature of the platform allowed for immediate feedback, creating a sense of Social Presence [14]. Consumers felt empowered when streamers responded to their specific requests, transforming a passive viewing experience into an active, game-like interaction.

Participant 02 described the excitement of having her specific request honored:

I commented ‘Show me number 3’, and he picked it up instantly. That feeling that he is listening to me right now makes the experience exciting.

This responsiveness fosters a psychological state of enjoyment and immersion, often referred to as Flow [29]. The collective excitement of flash sales and crowd participation further amplifies this positive affect. Therefore, we propose:

Hypothesis 3: Real-time interactivity has a positive impact on consumers’ perceived enjoyment.

4 Rural Support Consciousness and the ‘Warm-glow’ Effect

A unique finding of this study is the emergence of Rural Support Consciousness. Extending the general concept of community awareness, Chinese consumers exhibited a strong altruistic motivation to support farmers and rural revitalization. This pattern reflects the warm-glow theory [32], where the act of helping others generates intrinsic emotional utility.

Participants noted that seeing the authentic orchard background and the farmers’ labor triggered a moral satisfaction that enhanced their shopping pleasure. Participant 09 shared her emotional connection:

Seeing the farmer in the background sweating in the orchard made me feel emotional. I felt that my purchase wasn’t just for eating, but to help them sell their harvest before it rots.

Furthermore, this moral satisfaction significantly bolsters trust and reduces purchase anxiety. As Participant 05 emphasized:

Because he showed the texture so clearly, I felt a sense of relief and trust that I wouldn’t be scammed. It made me feel happy to help the farmers.

This implies that the agricultural context adds a layer of social meaning to the consumption process, converting it from a mere transaction into a charitable act that significantly boosts perceived enjoyment and trust.

This implies that the agricultural context adds a layer of meaning to the consumption process, converting it from a transaction into a charitable act, which significantly boosts perceived enjoyment. Thus, the following Hypothesis is reasonable:

Hypothesis 4: Rural support consciousness has a positive impact on consumers’ perceived enjoyment.

5 The Mechanism of Impulse Buying: Trust and Enjoyment

Finally, the qualitative data suggested that the environmental stimuli (S) do not solely drive behavior; rather, they operate through the organismic states (O) of trust and enjoyment.Participants indicated that once trust was established (via visual freshness and expertise), the psychological barrier to purchase was removed, allowing them to act on impulse. Similarly, the heightened state of enjoyment (via interactivity and rural support) reduced their self-regulation.

As Participant 16 noted:

Watching the live stream is like a game show. When he interacts with us, I feel happy and excited... and before I know it, I’ve clicked ‘buy’.

This narrative confirms the mediation logic of the S-O-R framework in this context, leading to the final set of hypotheses:

Hypothesis 5: Perceived trust positively influences impulse buying intention.

Hypothesis 6: Perceived enjoyment positively influences impulse buying intention.

### 4.2. Phase 2: Quantitative results

#### 4.2.1. Sample profile.

The presents the sample profile based on all valid surveys (N = 428). About two-thirds (62.4%) of the surveyed consumers were women. The percentage of female consumers exceeded that of men, reflecting a reality wherein women often act as the primary decision-makers for household fruit and grocery consumption in China.

Regarding age, 78.5% of the respondents were aged between 25 and 40. This agricultural live streaming content thus seems to appeal primarily to young and middle-aged adults (digital natives) who possess the technological proficiency to navigate the fast-paced interactive interface of Douyin. About three-quarters (72.2%) of the participants held a bachelor’s degree or higher; these individuals likely possess the necessary digital literacy to engage in real-time interactions and flash sales.

Finally, fresh durians are considered luxury fruits with a high unit price in the Chinese market. Consumers usually require sufficient financial standing to support such hedonic consumption. More than half (54.4%) of the respondents earned a monthly household income greater than 10,000 RMB, indicating a relatively affluent consumer base (**S4 Table**).

#### 4.2.2. Measurement model assessment.

The measurement model was evaluated through two sequential steps. It was first necessary to examine reliability and convergent validity for the reflective constructs; the outer loading indicators, composite reliability (CR), and average variance extracted (AVE) should be ≥ 0.7, ≥ 0.7, and ≥ 0.5, respectively [43]. **S4 Table** shows that all loading indicators exceeded 0.708 (see Appendix for full cross-loading matrix), AVE values ranged from 0.742 to 0.853 (greater than 0.5), and CR values ranged from 0.909 to 0.946 (greater than 0.7). Furthermore, Cronbach’s alpha (α) values for all constructs were above 0.85. Reliability and convergent validity were hence adequate for the sample (**S5 Table**).

Discriminant validity was assessed next. Hair et al (2017) stated that acceptable heterotrait-monotrait (HTMT) values should be less than either 0.85 or 0.90; we applied the more rigorous conservative threshold of 0.85 [45]. **S5 Table** indicates that discriminant validity was acceptable across all constructs, with the highest HTMT ratio being 0.450 (between Perceived Trust and Visual Freshness), which is well below the threshold. This confirms that the distinct dimensions of the agricultural e-servicescape (e.g., visual freshness vs. streamer expertise) and the organismic states are empirically distinct (**S6 Table**).

### 4.3. Evaluating the structural model

The structural model was evaluated to test the hypothesized relationships. Before analyzing the path coefficients, collinearity was examined; the Variance Inflation Factor (VIF) values for all predictor constructs were close to 1.0 (ranging from 1.000 to 1.005), indicating no multi-collinearity issues.

The presents the assessed structural model results. Product coefficients (conveying indirect relationships) were considered when evaluating the presence of a significant mediating effect based on bias-corrected bootstrap confidence intervals (5,000 sub-samples) [50]. The results indicate significant direct effects for all hypothesized paths. Specifically, Visual Freshness (β = 0.422, *p* < 0.001) and Streamer Expertise (β = 0.288, *p* < 0.001) significantly influenced Perceived Trust, supporting H1 and H2. The f2 effect sizes were 0.241 (medium) and 0.113 (small), respectively. Similarly, Real-time Interactivity (β = 0.321, *p* < 0.001) and Rural Support Consciousness (β = 0.307, *p* < 0.001) were significant predictors of Perceived Enjoyment, supporting H3 and H4 (small effect sizes). Both Perceived Trust (β = 0.222, *p* < 0.001) and Perceived Enjoyment (β = 0.203, *p* < 0.001) significantly drove Impulse Buying Intention (H5 and H6 supported) (**S7 Table**).

The mediation analysis confirmed that the organismic states fully mediate the relationship between the e-servicescape stimuli and impulse buying. Significant indirect effects were found for all paths (e.g., VF → Trust → IB, β = 0.094).

The explanatory power and predictive relevance of the model were evaluated using the coefficient of determination (R^2^), Q^2^, and PLSpredict. The model explained 26.1% of the variance in Perceived Trust (R^2^ = 0.261) and 19.1% in Perceived Enjoyment (R^2^ = 0.191). For the ultimate dependent variable, Impulse Buying Intention, the R^2^ was 0.084. While this value is relatively modest, it is statistically significant. Furthermore, the Q^2^ values for all endogenous constructs (Trust: 0.257; Enjoyment: 0.179; IB: 0.077) were greater than zero, confirming predictive relevance(**S2 Fig**).

To further validate the model’s out-of-sample predictive power, we performed the PLSpredict analysis. **S7 Table** shows that the PLS-SEM root mean squared error (RMSE) values were consistently lower than those of the Linear Model (LM) benchmark for all key target constructs (e.g., PLS RMSE for Impulse Buying = 0.960 vs. LM RMSE = 1.001). This confirms that the model possesses strong predictive power compared to a naive linear benchmark (**S8 Table**).

## 5. Discussion

The antecedents of impulse buying in live streaming commerce constitute an emerging and critical issue in agricultural marketing research, especially as they relate to platform-specific environmental cues and psychological mechanisms. A clearer understanding of this topic can advance knowledge of the screen-to-consumer relationship for high-value fresh produce. To achieve this study’s aims, a sequential mixed-methods design was employed to reshape the e-servicescape framework and investigate its impact on Chinese consumers’ impulse purchasing of fresh durians.

### 5.1. Theoretical integration and findings

The current findings generally correspond with and extend those of previous studies on live streaming commerce. A few scholars have explored the impact of general live streaming features on consumer engagement and trust building [1,3,14]. Recently, Teng and Balakrishnan (2025) enriched the retail e-servicescape through qualitative exploration. Similar to this earlier work, the findings of this study pinpointed the agricultural e-servicescape as a principal mechanism transmitting the effect of environmental stimuli to impulse buying intention [15].

However, this study sheds new light on the explanatory power of context-specific dimensions. While general attractiveness is often cited in retail literature [12], our qualitative and quantitative results confirmed that for fresh durians, this dimension is better conceptualized as Visual Freshness. The high path coefficient (β = 0.422) from Visual Freshness to Perceived Trust validates Product Diagnosticity Theory [16] in a novel context: when tactile inputs are missing, high-definition visual details (e.g., thorn texture, flesh color) serve as the primary diagnostic cues for quality, effectively reducing the risk.

Furthermore, the significant role of Streamer Expertise (β = 0.288) reinforces Source Credibility Theory [27]. Unlike standard influencers who rely on charisma, fruit streamers act as quality gatekeepers. Their demonstrated skill (e.g., knocking on the shell) acts as a trust transfer mechanism, which is essential for non-standardized agricultural products.

### 5.2. The psychological mechanism: Trust and enjoyment

Examining the mediators of Perceived Trust and Perceived Enjoyment is essential to understanding impulse buying. This study revealed that the agricultural e-servicescape indirectly affected impulse buying intention through these two organismic states, fully validating the S-O-R paradigm.

1 Trust as a Precondition:

Despite the impulse nature of the behavior, our findings suggest that cognitive trust is a prerequisite for high-value agricultural purchases. The strong link between trust and impulse buying (β = 0.222) implies that consumers first need a safety net (believing the fruit is fresh) before they can succumb to the urge to buy. This contradicts some views that impulse buying is purely emotional; in the context of durian blind boxes, it is a trust-enabled impulse.

2 Enjoyment as a Driver:

The study also confirmed that Perceived Enjoyment is a powerful driver (β = 0.203), fueled significantly by Real-time Interactivity and, uniquely, Rural Support Consciousness. The latter is a standout finding. Consistent with the warm-glow effect [32], Chinese consumers derive hedonic value from the altruistic act of helping farmers. Seeing the orchard background and believing their purchase aids rural revitalization enhances their shopping pleasure, which subsequently triggers impulse buying. This suggests that in the agricultural sector, moral satisfaction is a component of hedonic enjoyment.

### 5.3. Comparison with general E-commerce

Compared to traditional e-commerce products (e.g., apparel or electronics), the nuances of live streaming agricultural marketing explain the unique weight of our findings. In general e-commerce, website layout or system availability are often dominant constraints [25]. However, in fruit live streaming, sensory verification (Visual Freshness) and social connection (Rural Support) take precedence. The high variance explained in Perceived Trust (R2 = 26.1%) by these specific cues indicates that for high-risk perishable goods, the servicescape is not just a background setting but a dynamic evidence system for quality and ethics.Furthermore, compared to traditional offline fruit markets where consumers physically touch and smell the produce, live streaming compensates for this sensory absence by offering a hyper-focused, expertly guided visual inspection, transforming a physical limitation into a dynamic digital interaction.

## 6. Conclusion

### 6.1. Theoretical contributions

This study offers several significant theoretical contributions to the literature on agricultural marketing and live streaming commerce. First, although scholars have extensively explored impulse buying behavior employing multiple models linking website quality, trust, and enjoyment, the specific nuances of the agricultural e-servicescape have remained underexplored. Therefore, this work advances theory development by reshaping the traditional e-servicescape framework to explain the screen-to-consumer mechanism for high-value fresh produce. Findings offer support for the applicability of Product Diagnosticity Theory in live streaming by confirming that Visual Freshness (rather than general aesthetic appeal) serves as the primary diagnostic cue for trust in non-standardized goods. This theoretical refinement expands relevant knowledge by emphasizing that for perishable products, the servicescape functions as a quality verification system.

Second, this study uncovers the psychological mechanisms underlying the association between agricultural cues and behavioral response. By verifying the mediating roles of Perceived Trust and Perceived Enjoyment, our work promotes a deep understanding of the S-O-R in the context of durian blind boxes. Specifically, the results echo and extend the Source Credibility Theory by demonstrating that Streamer Expertise (e.g., knocking on shells) acts as a critical trust-transfer mechanism, effectively mitigating the information asymmetry inherent in the lemon market of fruits.

Third, a unique contribution of this study is the identification and empirical validation of Rural Support Consciousness as a key driver of hedonic enjoyment. No prior work has explicitly extended the e-servicescape model by incorporating this altruistic dimension within a commercial live streaming context. This contribution responds to prior calls for model extension using context-specific factors. By bridging the gap between ethical consumption (warm-glow effect) and impulse buying, this study reveals that for Chinese consumers, the act of helping farmers is not just a social responsibility but a source of shopping pleasure. Overall, this study replicates and extends the S-O-R framework through the lens of digital agriculture.

### 6.2. Practical contributions

These findings also provide several tangible benefits for practitioners such as fruit farmers, live streamers, and platform managers. Our results highlight Visual Freshness and Streamer Expertise as the strongest predictors of trust. This outcome is particularly relevant for live streaming hosts; those selling high-risk fruits like durians should prioritize high-definition, close-up demonstrations of product texture (e.g., pressing the thorns) over mere entertainment. Streamers must position themselves as quality gatekeepers by learning professional selection techniques and demonstrating candid rejection of inferior fruit live on camera. Doing so could boost consumers’ cognitive trust and reduce their perceived risk.

Moreover, this study identified Rural Support Consciousness as a significant driver of enjoyment. Agricultural sellers can thus enhance their content strategy by showcasing the Origin Authenticity, broadcasting directly from the orchard and highlighting the farmers’ labor. Storytelling that connects the purchase to rural revitalization can trigger the warm-glow effect, transforming a transactional purchase into an emotional experience.For platform managers (e.g., Douyin), the findings suggest that optimizing the interface to facilitate Real-time Interactivity (e.g., one-click response tools for streamers) is crucial. Since enjoyment mediates the impact of interactivity on impulse buying, platforms should design features that gamify the shopping process, thereby fostering a state of flow and sustainable sales growth.

Finally, regarding the broader implications for regional development projects such as the Digital-Intelligence Driven Development of Cultural Sojourn Industry, this study offers a transferable model. The verified impact of Visual Freshness and Rural Support suggests that to successfully market its cultural tourism and local specialties (e.g., tea, embroidery) online, it must build a high-fidelity, immersive digital servicescape. Policymakers and industry leaders can leverage these findings to train local practitioners in digital-intelligence storytelling, transforming local cultural assets into trust-building and enjoyment-inducing digital content, thereby achieving a sustainable development path driven by digital intelligence.These findings offer actionable strategies for the value chain reconstruction of emerging agricultural sectors (e.g., the import substitution of regional specialty industries). By leveraging the validated elements of visual freshness and rural support, agricultural practitioners can transition from low-margin bulk selling to premium, digital-intelligence driven brand building, ensuring sustainable market competitiveness.

### 6.3. Limitations and future research directions

Several limitations of this study illuminate avenues for further work. First, due to the specific focus on fresh durians,a high-unit-price, high-risk product,the results may not be fully generalizable to low-involvement agricultural products like potatoes or apples. Researchers can gather data on different fruit categories to cross-validate the reshaped e-servicescape dimensions in the future.

Second, we only considered Impulse Buying Intention as the final behavioral outcome; the findings of this study may not reflect the long-term effects of the agricultural e-servicescape, such as customer loyalty or continuous purchase intention. Subsequent work should explore these post-purchase behaviors to provide a more comprehensive picture of sustainable agricultural e-commerce.

Third, although PLS-SEM was employed for quantitative analysis, the cross-sectional nature of the survey data precludes strict causal inferences. Future studies could apply longitudinal designs or field experiments (e.g., A/B testing different live streaming scenarios) to reveal more robust causal relationships.

Last, this study focused on the Chinese market (Douyin). Given that live streaming commerce is a global phenomenon, the cultural elements of Rural Support Consciousness might differ in other countries. Subsequent studies should thus apply the current model to other cultural contexts (e.g., TikTok in Southeast Asia) to test the cross-cultural generalizability of the agricultural e-servicescape.

## Supporting information

S1 Fig The conceptual framework. (TIF)

S2 Fig Structural model results. (TIF)

S1 Table Operationalization of qualitative themes into quantitative survey items. (XLSX)

S2 Table Example of coding process. (XLSX)

S3 Table Coding scheme of the agricultural live streaming e-servicescape. (XLSX)

S4 Table Demographic profile of the respondents (N = 428). (XLSX)

S5 Table Measurement model assessment. (XLSX)

S6 Table Discriminant validity (HTMT). (XLSX)

S7 Table Structural model assessment (Direct and Indirect Effects). (XLSX)

S8 Table PLSpredict results. (XLSX)
