# Correction: From mobile phone screen to taste buds: A study on the impact of Douyin live streaming E-service scenarios on Durian consumer trust and purchase intention

**DOI:** 10.1371/journal.pone.0358564

**Published:** 2026-09-15

**Authors:** Xuemei Xu, Anita Rosli, Aryaty Alwie, Ismawati Sharkawi, Yurong Wu

The first author, Xuemei Xu, is incorrectly noted as the corresponding author. The correct corresponding author is Anita Rosli. Anita Rosli’s email address is: anitarosli@upm.edu.my.
